# Uncommon Encounter in Urachus: A Rare Case of Xanthogranulomatous Urachitis With Review of Literature

**DOI:** 10.1002/ccr3.71897

**Published:** 2026-01-21

**Authors:** Mohamed Javid Raja Iyub, P. Dhineshkumar, Fatima Saibeer Bagalkot, Aishwarya Katiah, Poorvikha S., Kainat Jahangir, Vivek Sanker, Tirth Dave

**Affiliations:** ^1^ Miami Cancer Institute Baptist Health South Florida Miami Florida USA; ^2^ Stanley Medical College Chennai India; ^3^ Gulf Medical University Ajman UAE; ^4^ Gandhi Medical College Hyderabad India; ^5^ St John's Medical College Bangalore India; ^6^ Faculty of Medicine Dow Medical College Karachi Pakistan; ^7^ Department of Neurosurgery Stanford University Stanford California USA; ^8^ Bukovinian State Medical University Chernivtsi Ukraine

**Keywords:** chronic inflammation, urachal xanthogranuloma, urachus, xanthogranulomatous urachitis

## Abstract

Urachus is a fibrous remnant from embryologic development derived from the allantois, which is involved in waste elimination in the fetus. Typically, it extends from the dome of the bladder to the umbilicus. Xanthogranulomatous urachitis, or xanthogranulomatous inflammation of the urachus, is a highly unusual pathological entity characterized by large lipid‐laden macrophages, with only a few cases reported worldwide. In this case, a 48‐year‐old male patient presented with complaints of persistent abdominal pain and watery discharge from the umbilicus for 1 month. Following en bloc resection of the urachal mass, histopathology revealed findings consistent with xanthogranulomatous urachitis. The postoperative course was uneventful, and eventually, the patient recovered to baseline health with resolution of bothersome symptoms. We report a case of xanthogranulomatous urachitis, which, despite its rarity, should be considered an important differential diagnosis of all urachal masses.

## Key Clinical Message

Xanthogranulomatous urachitis, though rarely encountered in clinical practice, is an important differential diagnosis for urachal cancers owing to similar radiological imaging findings.

## 1 Introduction

Urachal xanthogranuloma or xanthogranulomatous inflammation of the urachus is infrequent, with only a few reported cases worldwide [1]. The exact etiology of the xanthogranulomatous lesions remains elusive. It has been suggested that xanthogranulomatous cystitis could result from chronic urachal cysts or diverticulum infections. Furthermore, an immunologic defect is hypothesized to play a role in developing xanthogranulomatous cystitis. Therefore, xanthogranulomatous inflammation of the urachus is also considered to have a similar origin and etiology [2]. Urachal cancers are far more common than the xanthogranulomatous inflammation of the urachus. The radiologic imaging finding of the xanthogranulomatous urachitis is similar to urachal cancer, hence warranting its inclusion in the differential diagnosis of any suspected mass arising from the urachus [2, 3]. This similarity further highlights the need for early surgical intervention to ensure the correct diagnosis. Herein, we present a 48‐year‐old male patient with discharge from the umbilicus for 1 month. Following en bloc resection, histopathologic examination (HPE) showed xanthogranulomatous inflammation of the urachus.

## 2 Case History/Examination

A 48‐year‐old male presented with a history of persistent abdominal pain and watery discharge from the umbilicus for 1 month. The patient had a history of recurrent urinary tract infections (UTIs) and experienced a similar episode 1 year prior. He did not have any other significant history. The patient was afebrile, and all other vital signs were normal. Examination revealed a vague, mildly tender mass of 5 × 4 cm below the umbilicus, which was firm in consistency and had ill‐defined margins. Laboratory investigations included a complete blood count, which reported an elevated white blood cell count (14,500/mm^3^). Urine culture was positive for *Escherichia coli* ( *E. coli* ) with a colony count of 10^5^ CFU/mL, which was sensitive to Amikacin and Meropenem. Accordingly, the patient was started on culture‐sensitive antibiotics. Renal function tests were within normal limits.

## 3 Methods

**FIGURE 1:**
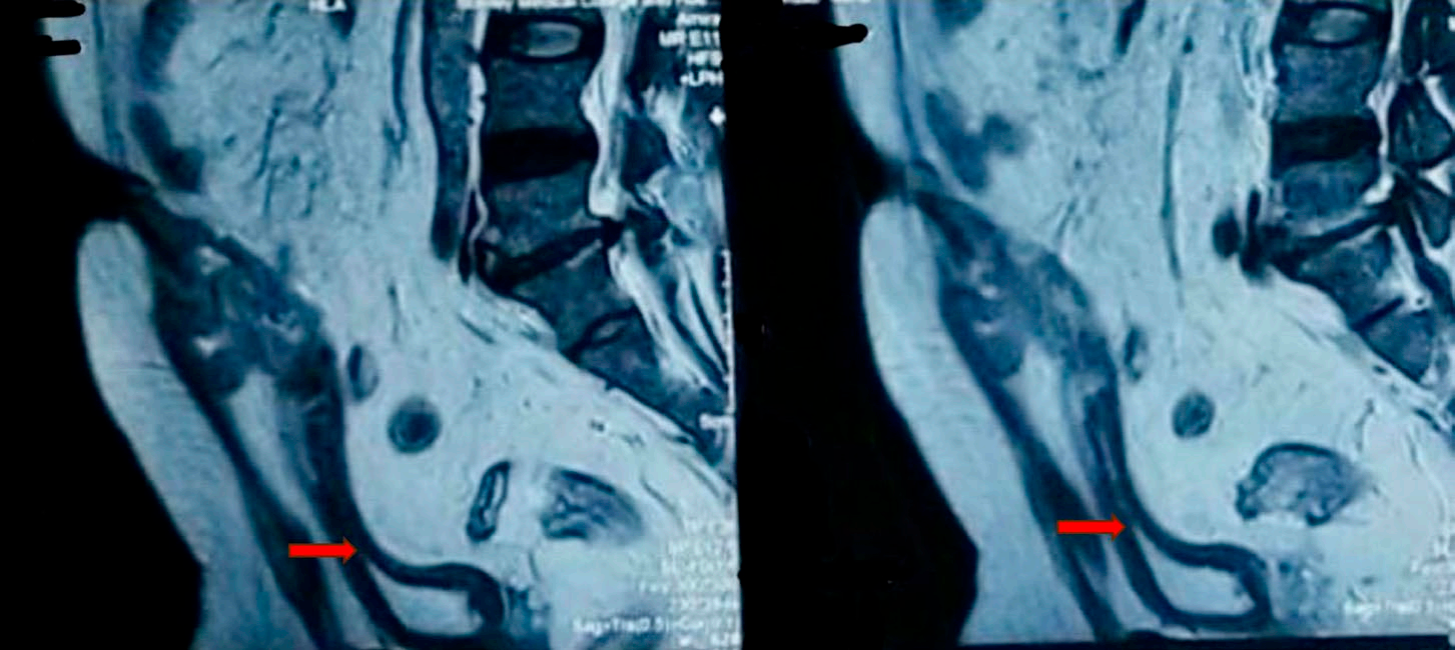
MRI showing an ill‐defined tract (red arrow) arising from the dome of the bladder, extending into the dermal plane, and opening into the umbilicus.

Ultrasound (US) of the abdomen and pelvis demonstrated a poorly defined, heterogeneously hypoechoic intra‐abdominal collection measuring 11 × 7 cm from the umbilicus to the superior margin of the bladder, with diffuse inflammatory changes. Abdominal computed tomography (CT) showed a peripheral, well‐defined, thick‐walled, heterogeneously enhancing hypodense lesion of size 8.8 × 5.6 × 11 cm noted in the umbilical region extending into the peritoneal cavity up to the dome of the urinary bladder. Surrounding fat stranding was also noted. Lymph node enlargements or metastatic lesions in the abdomen or pelvis were not suspected on imaging. These findings gave a preliminary impression of an infected urachal cyst with an abscess. Magnetic resonance imaging (MRI) demonstrated an ill‐defined tract approximately 7 cm in length originating from the dome of the bladder, extending into the dermal plane, and opening into the umbilicus (Figure 1). The tract abutted the rectus muscle and gave the impression of a possible urachal adenoma. Preoperative cystoscopy revealed a 5 mm puckering of the bladder mucosa on the bladder dome's right side. The rest of the bladder appeared normal.

**FIGURE 2:**
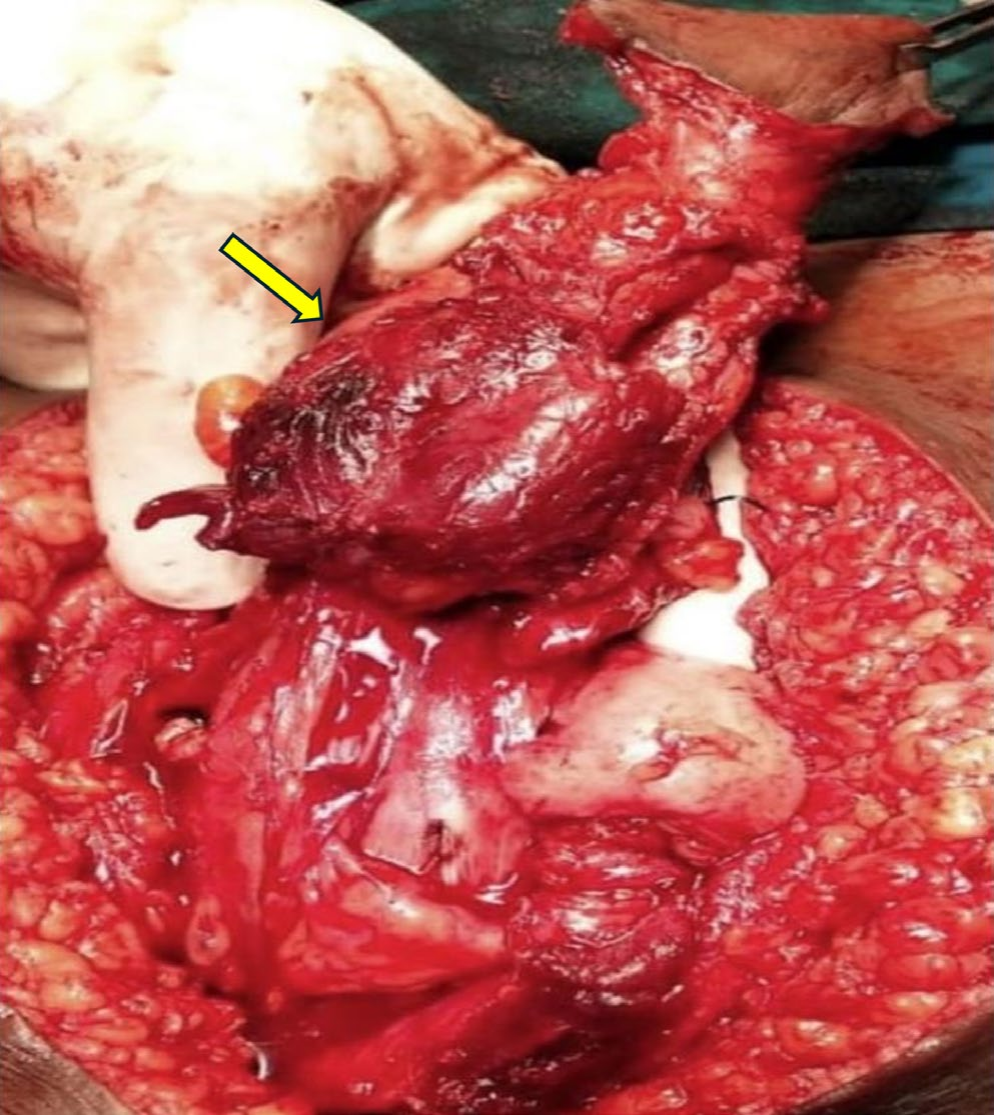
Intraoperative findings demonstrating a urachal tract with a firm mass (yellow arrow) in the middle of the tract.

Based on imaging and clinical findings, the patient underwent en bloc resection of the urachal mass with a cuff of the bladder and umbilicus. Intraoperative findings included a urachal tract measuring 10 cm in length, with a firm mass measuring 6 × 4 cm in the middle of the tract (Figure 2). The mass demonstrated inflammatory changes and was infiltrating the surrounding rectus muscle.

## 4 Conclusion and Results

**FIGURE 3:**
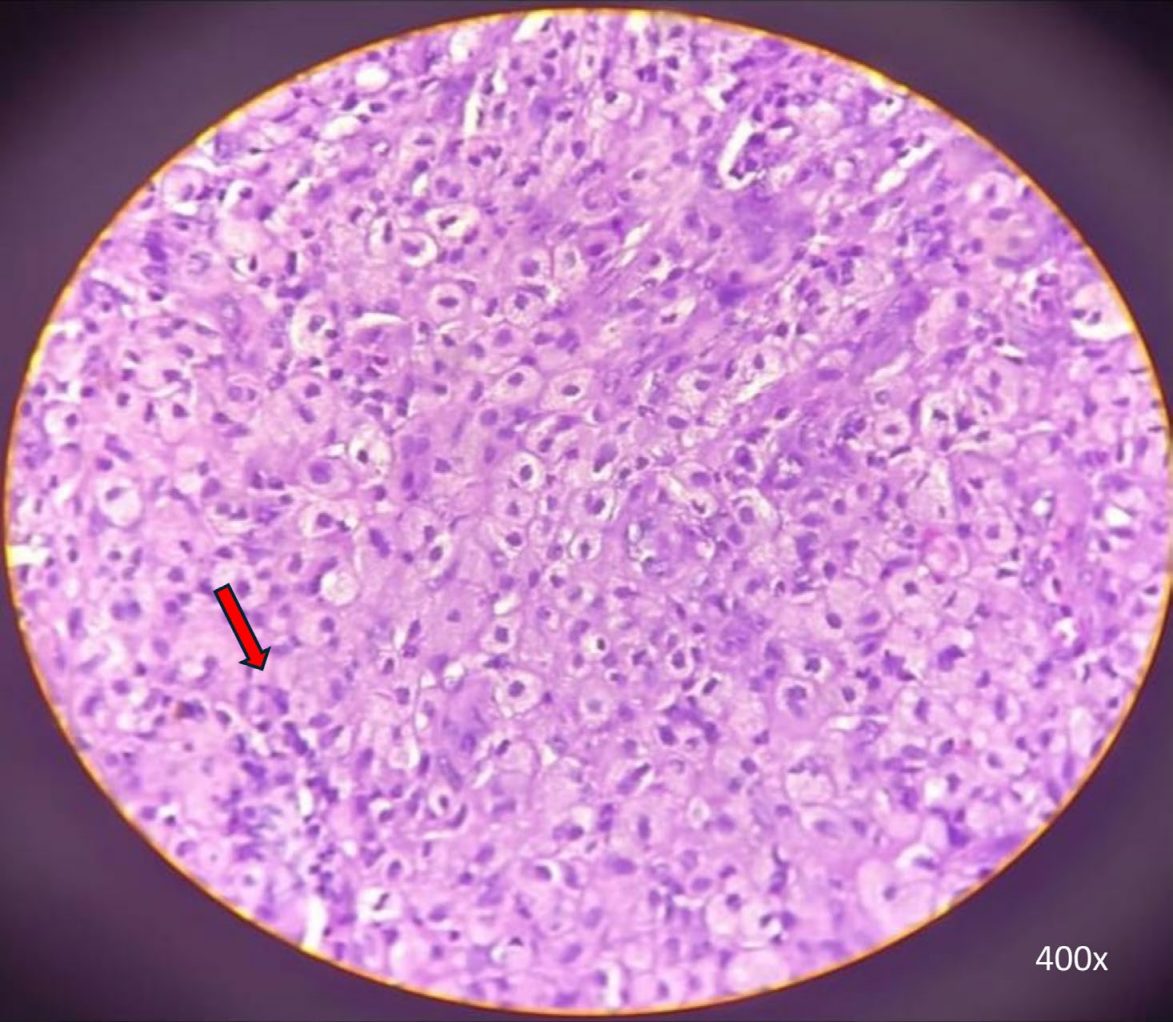
HPE revealing extensive infiltration of the urachal tissue by lipid‐laden foamy macrophages, multinucleated giant cells (red arrow), and lymphocytes (400× magnification).

Histopathological examination revealed extensive infiltration of the urachal tissue by lipid‐laden foamy macrophages, multinucleated giant cells, and lymphocytes (Figure 3). There was also evidence of fibrosis and chronic inflammation. These findings were consistent with xanthogranulomatous urachitis. The patient had an uneventful postoperative course and recuperated to his baseline health. Follow‐up of the patient at three and 6 months was completed, and all bothersome symptoms were resolved.

## 5 Discussion

Urachus is a fibrous remnant of the embryonic allantois, which connects the urinary bladder apex to the umbilicus and typically obliterates during fetal development. However, abnormalities can arise when this process is incomplete. Urachal abnormalities present a myriad of diagnostic challenges due to their rarity and diverse clinical presentations, as they often mimic many intra‐abdominal and pelvic diseases [4]. Typically, the urachus becomes non‐functional, giving rise to the median umbilical ligament. However, remnants of the urachus may persist, often presenting with symptoms in adulthood, among which malignancy is the most common cause. This was evident in a study conducted by Tian J et al., where among the 33 cases of urachal mass studied, 22 were found to be adenocarcinoma, whereas the remaining 11 were reported to be benign. Of all these, only one case was identified as xanthogranulomatous inflammation of the urachus [5].

Xanthogranulomatous inflammation is uncommonly characterized by large clusters of macrophages with lipid‐rich cytoplasm [6, 7]. It is predominantly observed in anatomical sites such as the kidney, ovary, retroperitoneum, stomach, etc. [6, 7]. However, its occurrence in the urachus is exceedingly rare but of immense clinical significance. Furthermore, only a few cases of xanthogranulomatous urachitis have been reported worldwide [1, 8]. According to a retrospective study, gross hematuria and age over 55 years increase the likelihood of a urachal mass being malignant by 17‐fold and 3‐fold, respectively [9, 10]. The absence of these features in our patient aligns with the probability of a benign mass. In our case, the patient experienced watery discharge from the umbilicus and abdominal pain, with examination demonstrating an infra‐umbilical mass. This presentation is similar to other case reports; however, our patient was afebrile.

US and CT are the preferred initial investigations in evaluating a urachal mass. However, differentiating urachal carcinoma from a xanthogranulomatous inflammation based on imaging alone can be challenging due to the complex echogenicity in the US and thick‐walled heterogeneous attenuation on CT, as noted in our patient [4, 8].

Due to the rarity of the pathology in this location, there are no established guidelines regarding the treatment for xanthogranulomatous inflammation of the urachus [1]. As performed in this case, surgical excision with en bloc resection of the urachal mass remains the standard treatment approach. However, the contemporary consensus in the management of urachal carcinoma is the surgical en bloc excision of the umbilicus, median umbilical ligament, also known as the urachal ligament, partial cystectomy, followed by removal of lymph nodes in the pelvic region (pelvic lymphadenectomy). Due to the preoperative challenges that exist in differentiating malignancy from urachal xanthogranuloma, a similar procedure is often undertaken in cases of urachal xanthogranuloma [2, 3]. However, as the significance of pelvic lymphadenectomy is not currently known in these cases and due to the associated morbidity of the procedure, it might not be needed in cases of xanthogranulomatous urachitis [2, 3]. Histopathological examination of the specimen can confirm the diagnosis and concurrently exclude a malignancy. A summary of the published cases of xanthogranulomatous urachitis is shown in Table 1.

**TABLE 1:** A summary of the cases of xanthogranulomatous urachitis in existing literature.

Sl. no	Title	Year	Author	Age/gender	Clinical features	Investigations	Management	HPE findings	Prognosis
1.	Urachal xanthogranuloma: a rare but important case presenting as a urachal mass	2023	Kunal Jain et al. [1]	55‐year‐old male	Three‐month history of left lower abdominal pain, urinary frequency, nocturia, and urgency	Abdominal ultrasound: 4.2 × 6.1 × 5.3 cm mass seen near the superior region of the urinary bladder Cystoscopy: unremarkable without any bulge at the dome CT (Computed tomography) abdomen/pelvis: 10.3 cm cystic and solid mass that abuts the superior aspect of the bladder and extends to the level of the umbilicus	Surgical excision of the mass and partial cystectomy with bilateral extended pelvic lymph node dissection	Microscopically, the mass was composed of mixed inflammatory cells with foamy macrophages	Postoperatively, the patient had an uncomplicated hospital course Follow‐up in 2 months: Return to the baseline health with resolution of urinary symptoms Follow‐up imaging did not reveal any mass or any other significant findings
2.	Xanthogranulomatous inflammation of urachus	2022	Yahya Ghazwani et al. [2]	65‐year‐old male	Right lower quadrant and mild suprapubic tenderness with suprapubic and paraumbilical fullness	CT scan‐necrotic soft tissue mass with irregular thick peripheral enhancement arising from bladder dome	Surgical excision of mass along with partial cystectomy and bilateral pelvic lymph node resection	Negative for malignancy and showed xanthogranulomatous inflammation of urachal mass	NA (Not Available)
3.	Xanthogranulomatous urachitis	2020	Senthil Kumar Aiyappan et al. [3]	44‐year‐old female	Dysuria and lower abdomen pain for the past 10 days. Clinical examination revealed tenderness in the lower abdomen. No mass was palpable	Ultrasound showed presence of solid mass lesion with cystic area in dome of the urinary bladder. Contrast‐enhanced computed tomography (CECT) revealed a solid lesion with heterogenous enhancement along with a cystic region in dome of the bladder projecting toward umbilicus. This lesion measured around 5.5 cm craniocaudal	Laparoscopic excision of the urachal mass with partial cystectomy under general anesthesia	Histopathology of the lesion revealed xanthogranulomatous inflammation of the urachus with no evidence of malignancy	Follow‐up at 12 months revealed no new symptoms
4.	Xanthogranulomatous inflammation of urachus	2013	Jeong Hyun Oh et al. [4]	18‐year‐old male	Lower abdominal pain, hematuria, dysuria	CT showed urachal mass with bladder invasion, which was suspected to be an urachal carcinoma or abscess	Exploratory laparotomy—excision of the urachus and partial cystectomy was performed by way of lower midline incision	Histopathological examination identified the mass as an urachal xanthogranuloma	NA
5.	Urachal Xanthogranulomatous Inflammation	2010	Shu‐Fen Tseng et al. [5]	67‐year‐old woman	Recurrent voiding, frequency and dysuria for 2 years	Cystoscopy revealed bladder mucosa inflammatory change at the dome. Abdominal CT: 5.67 × 3.89 × 3.37 cm mass above the bladder that extends to the umbilicus and was suspicious of urachal carcinoma	Urachal excision with partial cystectomy	Pathology: Chronic inflammatory cells plus multinucleated giant cells. The histology diagnosis was urachal xanthogranuloma	The patient remained well without any recurrent symptoms after 3 months of follow up
6.	Xanthogranulomatous inflammation of urachus mimicking urachal carcinoma	2009	Tricia Li Chuen Kuo et al. [6]	59‐year‐old female	Lower abdominal pain and a mass of 1‐month's duration. A suprapubic mass was palpable on abdominal examination	CT of the abdomen and pelvis was performed. A 2.3 × 3.3‐cm mass in the lower abdomen along with rim enhancement. The mass extended from the dome of the urinary bladder and involved the anterior abdominal wall, with adjacent fat stranding noted	Exploratory laparotomy—excision of the urachus, and partial cystectomy was performed	The final histologic examination showed xanthogranulomatous inflammation	Follow‐up at the outpatient clinic: Except for a small wound hematoma, the patient was otherwise well
7.	Urachal Xanthogranuloma: Laparoscopic Excision with Minimal Incision	2009	Sungchan Park et al. [7]	23‐year‐old man	Severe lower abdominal pain and voiding frequency that had appeared 20 days prior to presentation. Physical examination identified diffuse hardness and tenderness without change of skin color in the lower abdominal midline area	CECT of the abdomen and pelvis: a soft tissue lesion around 4.9 cm in diameter was attached to the anterosuperior area of the urinary bladder	Preoperative cystoscopy: Diffuse inflammatory mucosal changes on the anterior baldder wall. Laparoscopic partial cystectomy and excision of the urachal remnant was performed	Histologic examination: The urachal mass had an urachal cyst with xanthogranulomatous inflammation & chronic peri vesical inflammation and fibrosis. The lesions revealed classic lipid‐laden foamy histiocytes favoring xanthogranulomatous inflammation	Patient didn't have any symptoms post 10 months of surgery
8.	Combined Xanthogranulomatous Urachitis and Bullous Cystitis	2008	Ji Eun Kwak et al. [8]	31‐year‐old female	Abdominal pain, urinary frequency and dysuria for 3 months. Physical examination detected a palpable mass in the suprapubic area	Cystoscopic examination: Bullous protrusions of the urinary bladder mucosa. MRI pelvis: single cystic mass with peripheral enhancement	Urachus, including the mass and a cuff of the bladder dome, was resected	Microscopically, the cyst contents consisted of xanthogranulomatous exudate. The xanthogranulomatous inflammation involved the bladder wall in full thickness along with peri vesical fat tissue; this extended up to mucosal surface, causing xanthogranulomatous bullous cystitis with surface erosion. Ultrastructural examination: Histiocytes revealed multiple electron lucent droplets without limiting membrane	NA
9.	Urachal abnormalities	2008	Nimmonrat A et al. [9]	12‐year‐old female	Fever, dysuria, lower abdominal pain for 2 weeks with mild tenderness	US: mixed echogenic mass in the anterior superior portion of the bladder with apparent intravesical extension CT: midline ill‐defined heterogeneously enhanced mass extending from umbilicus to the dome of bladder	At surgery mass was adherent to the sigmoid colon and terminal ileum. Excision of the mass with partial cystectomy along with ileal resection (10 cm) and sigmoidectomy (10 cm) was performed	Chronic xanthogranulomatous inflammation with fibrosis	No complications
				38‐year‐old male	Presented with an abdominal mass for one‐month Physical examination‐large tense cystic mass in the lower abdomen	US: large cystic mass in the lower abdomen. CT: large well defined thick‐walled cystic mass with enhancement. This extended from umbilicus to lower pelvis and was adherent to the dome of the urianry bladder	14 cm cystic mass was removed with partial cystectomy	Xanthogranulomatous inflammation.	Postoperative was uneventful
10.	A case of urachal xanthogranuloma suspected to be a urachal tumor	2004	Tomomasa Yamamoto et al. [10]	47‐year‐old female	Lower abdominal pain and fever. There was a palpable mass in the lower abdomen	US and CT: a cystic mass from the superior aspect of the bladder dome extending to the umbilicus MRI: T1 weighted imaging revealed mass with contrast enhancement	Resection of the urachus with partial cystectomy	Histological diagnosis: Xanthogranuloma	NA
11.	A case of urachal xanthogranuloma containing a calculus: CT and MRI findings	2004	Mayumi Takeuchi et al. [11]	66‐year‐old woman	Presented to the hospital with hematuria. Clinical examination revealed a hen's egg‐sized mass palpable in the hypogastric region, but she remained afebrile and asymptomatic except for the abnormal urinalysis including massive hematuria and pyuria	Excretory urography demonstrated a calculus with concentric laminations situated just above the apex of the urinary bladder. Unenhanced CT revealed a midline‐situated supravesical soft tissue attenuation cavitary mass containing a calculus. Low attenuation speckles were observed in the right wall of the mass CECT: strong enhancement of the mass. Adjacent bladder wall was thickened with perilesional infiltration Sagittal MRI: Mass found to be in continuity with the umbilicus T2‐weighted imaging: A hypointense calculus and minimal fluid collection in the cavity of the mass T1‐weighted imaging: Hyperintense speckles observed on the right wall of the mass that corresponded with the low attenuation speckles seen on unenhanced CT	Cystoscopy demonstrated an orifice on the vesical dome with pus discharge consistent with vesicourachal diverticulum. Removal of the mass with urachal remnant and vesical dome was performed	Pathological examination revealed chronic inflammatory reaction with abundant lipid‐laden macrophages. The pathologic diagnosis was urachal xanthogranuloma. Chemical analysis revealed that the calculus was composed of calcium oxalate and calcium phosphate suggesting its urinary origin	NA
12.	Urachal xanthogranuloma caused by a swallowed fish bone: a case report	2001	Yoshiaki Kinebuchi et al. [12]	30‐year‐old female	Bladder irritability and with development of high fever. Physical examination revealed a tender mass in the suprapubic area	CT scan and MRI indicated a cystic mass above the bladder dome, extending toward the umbilicus	Urachal abscess was suspected, and the mass was excised en bloc with the urachus. The wall of the mass was thickened, and a linear foreign body was detected in the mass, which was considered to be a fish bone	Pathological diagnosis of the mass was xanthogranuloma. A speculation that a swallowed fish bone had penetrated the bowel and might have migrated into the urachal cyst, which induced a xanthogranulomatous change of the wall	NA
13.	A case of urachal xanthogranuloma causing recurrent intestinal obstruction	2001	Toshinori Kasai et al. [13]	68‐year‐old man	Lower abdominal pain and fever. There was a tender mass palpable in the lower abdomen	Plain abdominal X‐ray film revealed multiple air‐fluid levels with dilated small bowel loops, suggesting intestinal obstruction. Abdominal US, CT and MRI revealed a solid mass extending from umbilicus to the bladder dome beneath the rectal muscle	There was normal mucosa of the bladder by cystoscopic examination. A urachal tumor was clinically suspected and en bloc removal of the mass, the remaining urachus, umbilicus, omentum and bladder dome was performed	The histological diagnosis was urachal xanthogranuloma	The patient remained in good health without any recurrence for 6 months since the surgery
14.	Xanthogranulomatous Urachitis: CT Findings	1998	M. J. Díaz Candamio et al. [14]	42‐year‐old male	Presented with 3‐month history of suprapubic pain, occasionally concurrent with fever	US: a 4 cm heteroechoic mass in the dome of the bladder. Cystography: progressive tapering of the bladder dome CT: 4.2 cm round mass found in the urinary bladder dome Unenhanced CT: Soft tissue attenuation mass with some punctate calcifications. CECT: Heterogeneous enhancement of the mass	Surgical excision confirmed the mass originated from the supravesical aspect of the urachus and extended into the urinary bladder dome	Microscopic examination: Chronic inflammatory reaction the urachal mass was primarily comprised of lipid‐laden macrophages with some lymphocytes. Histopathologic diagnosis was xanthogranulomatous urachitis	NA
15.	Urachal xanthogranulomatous disease – case report	1996	W. Carrere et al. [15]	75‐year‐old female	Abdominal pain of one year. 3 kg weight loss in 2 months. Examination: Tender mass in the hypogastric region that adhered to the deep structures. There were no other features of acute inflammation	Abdominal US and CT: 6.9 × 4.2 × 5.5 cm mass with a tubular structure that extended from the umbilicus to the urinary bladder dome. Radiological features were suggested an urachal carcinoma	‘En bloc’ resection of the mass, the urachus, umbilicus, omentum, peritoneum, posterior rectal fascia and urinary bladder dome was done	Histopathology: Chronic inflammatory reaction with extensive areas predominantly of macrophages with lipid‐rich cytoplasm. No malignant tissue was observed. The intense xanthogranulomatous inflammation involved omentum, urachal remnants and umbilicus	Postoperative course: uneventful Follw‐up at 1 year: Patient remained asymptomatic. CT abdomen: Normal

## 6 Conclusion

In conclusion, while rare, xanthogranulomatous inflammation of the urachus is an important diagnosis to consider in patients presenting with umbilical region abnormalities with relevant presentation. As seen in our patient and many other case reports, differentiating benign urachal lesions from urachal carcinoma based on imaging studies is challenging and hence requires surgical intervention followed by histopathology confirmation.

## Author Contributions

**Mohamed Javid Raja Iyub:** conceptualization, writing – original draft, writing – review and editing. **P. Dhineshkumar:** conceptualization, writing – original draft, writing – review and editing. **Fatima Saibeer Bagalkot:** writing – original draft, writing – review and editing. **Aishwarya Katiah:** writing – original draft, writing – review and editing. **Poorvikha S.:** writing – original draft, writing – review and editing. **Kainat Jahangir:** writing – original draft, writing – review and editing. **Vivek Sanker:** supervision, writing – original draft, writing – review and editing. **Tirth Dave:** writing – original draft, writing – review and editing.

## Funding

The authors have nothing to report.

## Consent

Written informed consent has been obtained from the patient to publish this case report and accompanying images.

## Conflicts of Interest

The authors declare no conflicts of interest.

## Data Availability

The data that support the findings of this study are available from the corresponding author upon reasonable request.
